# Laparoscopic versus open rectal resection: a 1:2 propensity score–matched analysis of oncological adequateness, short- and long-term outcomes

**DOI:** 10.1007/s00384-021-03841-w

**Published:** 2021-01-22

**Authors:** Giovanni Maria Garbarino, Giulia Canali, Giulia Tarantino, Gianluca Costa, Mario Ferri, Genoveffa Balducci, Emanuela Pilozzi, Giammauro Berardi, Paolo Mercantini

**Affiliations:** 1 Department of Medical Surgical Science and Translational Medicine, Sapienza University of Rome, Sant’Andrea Hospital, Via di Grottarossa 1035-39, 00189 Rome, Italy; 2 Department of Clinical and Molecular Medicine, Pathology Unit, Sapienza University of Rome, Sant’Andrea Hospital, Via di Grottarossa 1035-39, 00189 Rome, Italy

**Keywords:** Rectal cancer, Laparoscopy, Oncological adequateness, Short-term outcomes, Long-term outcomes

## Abstract

**Background:**

Laparoscopic resections for rectal cancer are routinely performed in high-volume centres. Despite short-term advantages have been demonstrated, the oncological outcomes are still debated. The aim of this study was to compare the oncological adequateness of the surgical specimen and the long-term outcomes between open (ORR) and laparoscopic (LRR) rectal resections.

**Methods:**

Patients undergoing laparoscopic or open rectal resections from January 1, 2013, to December 31, 2019, were enrolled. A 1:2 propensity score matching was performed according to age, sex, BMI, ASA score, comorbidities, distance from the anal verge, and clinical T and N stage.

**Results:**

Ninety-eight ORR were matched to 50 LRR. No differences were observed in terms of operative time (224.9 min. vs. 230.7; *p* = 0.567) and postoperative morbidity (18.6% vs. 20.8%; *p* = 0.744). LRR group had a significantly earlier soft oral intake (*p* < 0.001), first bowel movement (*p* < 0.001), and shorter hospital stay (*p* < 0.001). Oncological adequateness was achieved in 85 (86.7%) open and 44 (88.0%) laparoscopic resections (*p* = 0.772). Clearance of the distal (99.0% vs. 100%; *p* = 0.474) and radial margins (91.8 vs. 90.0%, *p* = 0.709), and mesorectal integrity (94.9% vs. 98.0%, *p* = 0.365) were comparable between groups. No differences in local recurrence (6.1% vs.4.0%, *p* = 0.589), 3-year overall survival (82.9% vs. 91.4%, *p* = 0.276), and disease-free survival (73.1% vs. 74.3%, *p* = 0.817) were observed.

**Conclusions:**

LRR is associated with good postoperative results, safe oncological adequateness of the surgical specimen, and comparable survivals to open surgery.

## Introduction

Colorectal cancer (CRC) is the third most common cancer worldwide and the fourth cause of cancer-related deaths. About 35% of CRC are localized in the rectum [1]. Nowadays, the treatment of CRC is based on a multidisciplinary approach which combines surgery, chemotherapy, and radiotherapy. Whenever applicable, curative-intent surgery represents the foundation of CRC as it allows for the complete removal of the tumour and the accurate staging of the disease. Complete excision of the mesorectum containing the lymph nodes and the tumour represents the gold standard to achieve a radical oncological resection and reduce the chance of recurrence within the pelvis [2, 3]. Surgery is considered oncologically adequate if a clear distal margin and a circumferential radial margin wider than 1 mm are respected, and a complete mesorectal integrity is obtained [4]. As a matter of fact, patients who benefit from an oncologically adequate resection have an improved local control of the disease and a better long-term survival [2, 4, 5].

In an effort to minimize the impact of surgery, minimally invasive techniques such as laparoscopy and robotics have been implemented in the last 30 years. In this setting, laparoscopic resection for right and left colon cancer has been associated with better short-term outcomes and similar survivals as compared to open [6–12]. Notwithstanding, laparoscopic resection for rectal cancer (LRR) has not been validated yet by international guidelines, unless it is being performed in high-volume centres by experienced surgeons [1, 3]. Indeed, the rate of conversions reported in the literature is still high, as well as the involvement of the circumferential resection margin [13–15]. The complexity of the pelvic anatomy, the need for a complete excision of the mesorectal fascia, and the clearness of the circumferential margin make minimally invasive rectal surgery much more difficult than open, especially considering lower tumours in a narrow pelvis. Despite this, supporters of the laparoscopic approach speculate that oncological adequateness could still be maintained with safe recurrence rates and survivals, with the benefit of improved short-term outcomes [16, 17].

Due to the lack of consensus, the aim of this study was to compare laparoscopic and open resections for rectal cancer in terms of the oncological adequateness of the surgical specimen and to evaluate the perioperative and oncological outcomes.

## Methods

**Fig. 1:**
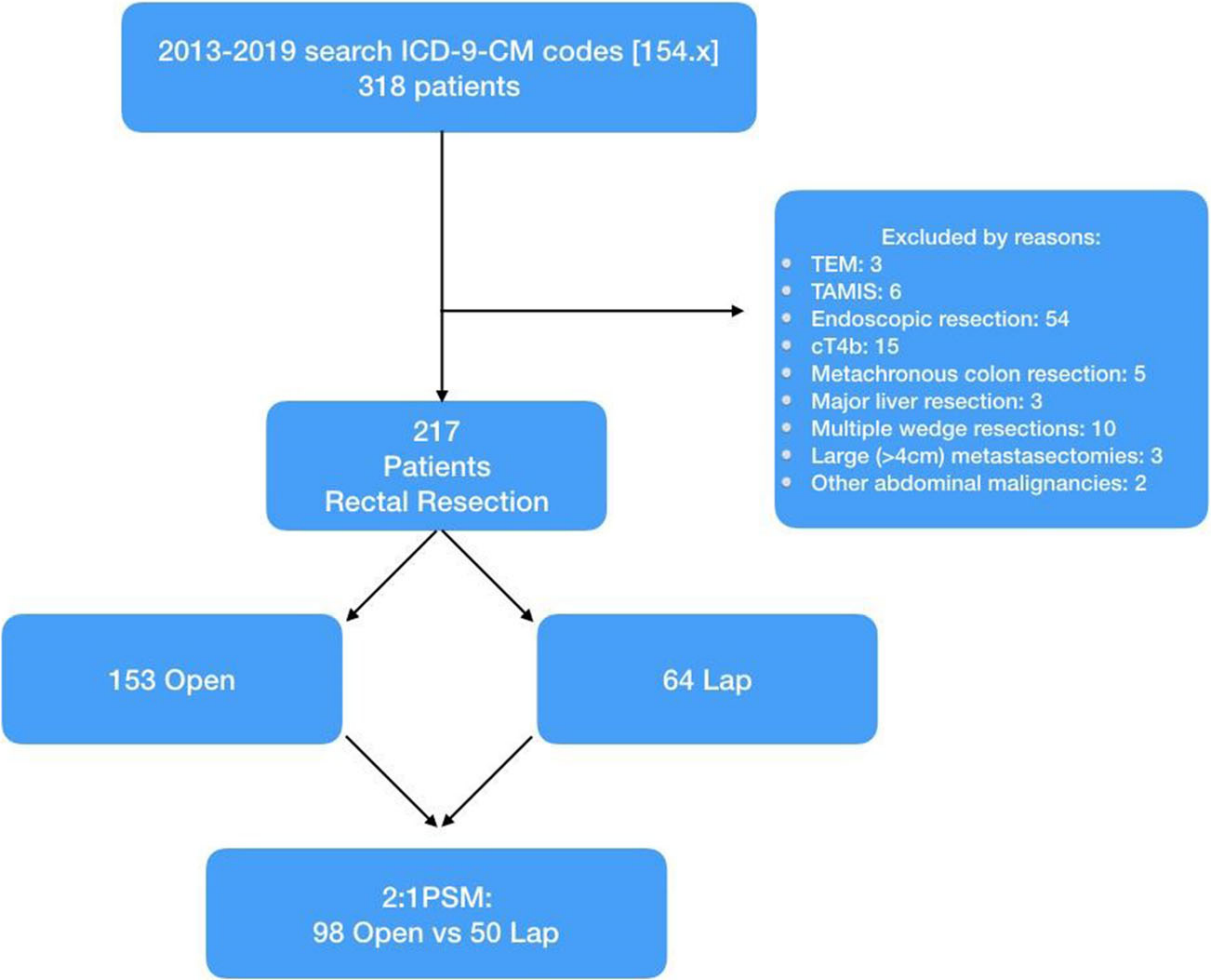
Flow chart of clinical study design (PSM, propensity score matching)

All consecutive patients undergoing surgery for rectal cancer at the General Surgery Department of Sant’Andrea University Hospital from January 1, 2013, to December 31, 2019, were included in this study. Data were retrospectively reviewed from a prospectively maintained database including demographics, tumour characteristics, operative details, tumour pathology, and short- and long-term outcomes. All patients aged 18 or older, with a histological diagnosis of adenocarcinoma of the rectum within 15 cm from the anal verge who underwent a rectal resection, were included in the study. Early rectal cancers treated by endoscopic resection, transanal endoscopic microsurgery (TEM), or transanal minimally invasive surgery (TAMIS); advanced cancers invading surrounding structures, metachronous tumours, metastatic cancers requiring major hepatectomies or more than two metastasectomies; patients with a single hepatic metastasis larger than 4 cm as well as patients with other abdominal malignancies were excluded. Patients were then divided into two groups according to the surgical approach: laparoscopic (LRR group) and open (ORR group) (Fig. 1).

No formal protocol for the allocation of patients to either group was established. The exclusion criteria for the LRR group were medical contraindication to the pneumoperitoneum, hostile abdomen preoperative predictive score ≥ 3, [18] previous surgery for endometriosis, previous gastric bypass for obesity, and patient’s choice.

The primary endpoint of our study was the oncological adequateness of the resected specimen. As secondary endpoints, perioperative morbidity and mortality and long-term oncological outcomes were evaluated and compared between groups.

All patients were discussed in a multidisciplinary environment involving surgeons, medical oncologists, radiologists, and pathologists. A formal Institutional Review Board approval was not required because of the non-interventional retrospective design; however, a signed consent for treatment and for the analysis of data for scientific purposes was obtained from all patients before surgical intervention.

### Definitions and study criteria

Preoperative clinical staging was assessed by pelvic magnetic resonance imaging (MRI) or endorectal ultrasound in all patients and a total body CT scan was performed in order to detect distant metastases.

**Fig. 2:**
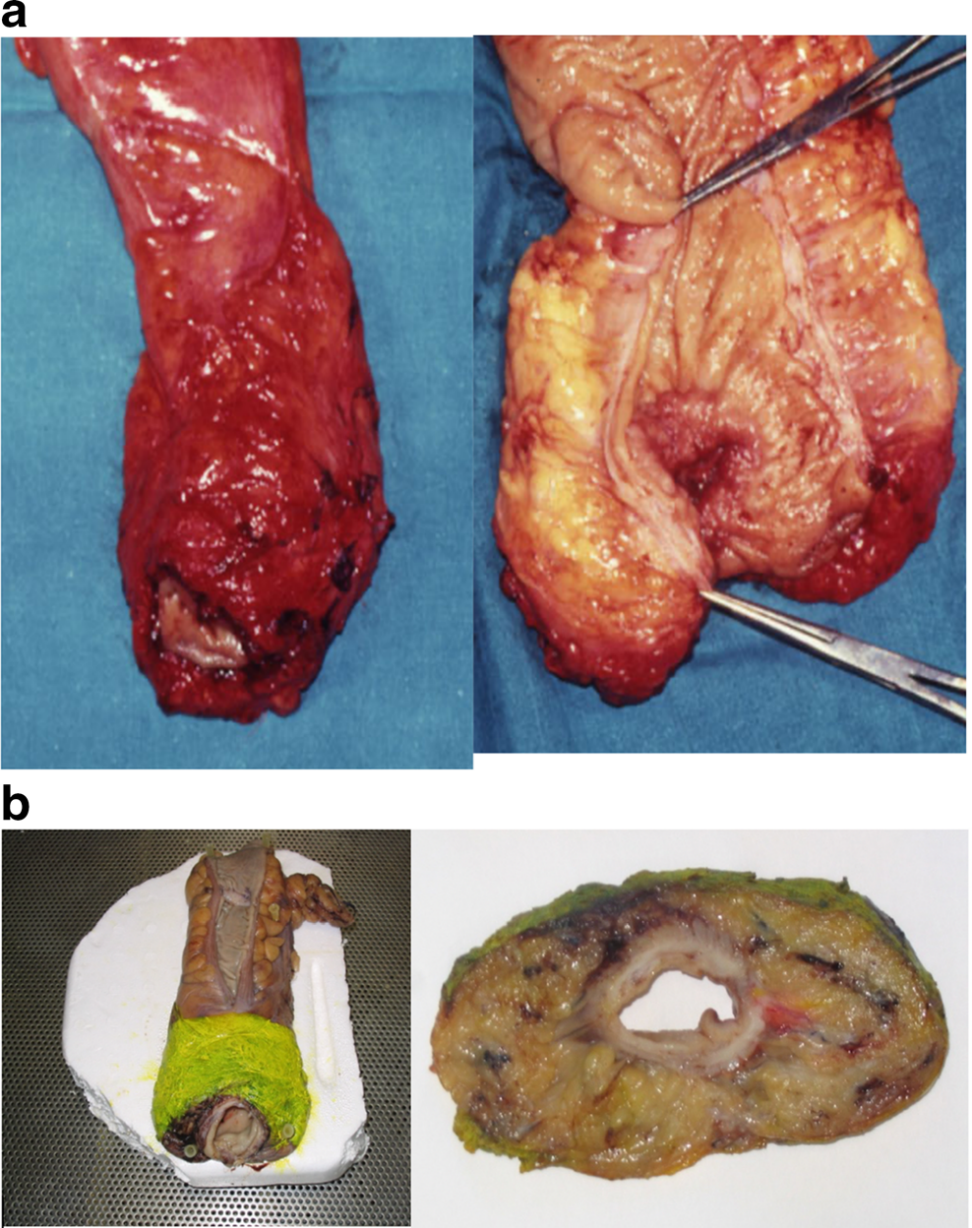
(A) Complete integrity of the mesorectal fascia; (B) the inked specimen of a total mesorectal excision (TME) and the cross-sectional slice for the evaluation of circumferential resection margin (CRM)

Cancers were defined as high (10–15 cm), middle (5–10 cm), or low (0–5 cm) rectal tumours according to the distance from the anal verge. Surgical specimens were examined by a single pathologist (E.P). These were received fresh and opened on the anterior wall from the proximal margin up to 4 cm above the peritoneal reflection for fixation. Before sampling, the integrity of the mesorectum was assessed and the circumferential margin below the peritoneum was inked (Fig. 2). The specimen was then sliced at 1-cm intervals starting from the distal margin. Tumour distance from the circumferential margin was recorded, both macroscopic and microscopic. Lymph nodes close to the circumferential margins were sampled separately. The oncological adequateness of the surgical resection was defined by the contemporary achievement of the following three parameters: (1) a distal resection margin ≥ 1 mm, (2) a circumferential radial margin (CRM) ≥ 1 mm, and (3) a complete mesorectal fascia integrity. Large defects (> 5 mm) of mesorectal fascia were classified as incomplete mesorectal excision. Staging was reported according to the 8th TNM edition [19].

Morbidity and mortality were defined as postoperative complications and death within 30 days from surgery respectively. Morbidity was graded according to the Clavien-Dindo classification and complications graded > II were defined as major [20].

Overall survival (OS) was defined as the time between surgery and death for any cause or last follow-up. Disease-free survival (DFS) was defined as the time from the operation to tumour recurrence either local or distant.

Neoadjuvant long- or short-course chemo-radiotherapy and adjuvant chemotherapy were administered according to the guidelines of the Italian Society of Medical Oncology (AIOM).

### Surgical technique

Open procedures were performed through a midline laparotomy while a 3 to 4 trocar technique with Pfannesteil incision for the specimen extraction was used in the laparoscopic group. A central ligation of the inferior mesenteric vessels was carried out in both open and laparoscopic cases and the splenic colonic flexure was always taken down. The lateral-to-medial technique was usually preferred in open procedures, whereas medial-to-lateral was more often chosen in laparoscopic surgeries. All the procedures were performed respecting the principles of the total mesorectal excision (TME) [2]. Partial mesorectal excision (PME) was carried out for high rectal tumours whenever a lower margin ≥ 5 cm was anticipated, avoiding coning resection lines. Diverting or terminal ostomies were reserved for patients with extraperitoneal rectal cancers who underwent neoadjuvant treatment. An end-to-end colorectal Knight-Griffen anastomosis was performed both in ORR and LRR cases when intestinal restoration was decided. All surgical procedures both open and laparoscopic were performed by four senior and experienced surgeons.

### Statistical analysis

Continuous data were expressed as the mean (± standard deviation) or median and interquartile range depending on their distribution that was assessed through the Shapiro-Wilk test. An unpaired Student *t* test was used to compare differences in continuous parametric variables and the Mann-Whitney *U* test for continuous non-parametric variables. Numbers and percentages were used for reporting categorical variables and the *χ*^2^ test was used for comparisons. A propensity score matching was applied to eliminate selection bias between groups and reported according to the recommendations of Lonjon et al. [21] Variables influencing decision regarding surgical approach and variables with potential influence on outcomes were assigned propensity scores using a multivariable logistic regression model. The final model included the following variables: age, sex, BMI, ASA score, comorbidity, distance from the anal verge, and clinical T and N stage. Setting a calliper width of 0.3, cases were matched to controls without replacement to the closest matched propensity score with a 1:2 ratio. Laparoscopic procedures converted to open or completion of any part of the pelvic dissection through the site of the specimen extraction were examined according to a per-protocol analysis and included in the ORR group.

Survival analyses were conducted using the Kaplan-Meier method with log-rank test comparisons. Significance was defined as a *p* value less than 0.05. Statistical analysis was performed using the SPSS software 25.0 (SPSS, Inc., Chicago, IL).

## Results

Between January 2013 and December 2019, a total of 318 patients underwent a rectal resection for a histologically proven adenocarcinoma at our institution. Three patients who underwent TEM, 6 TAMIS, 54 endoscopic resections, 15 resections of surrounding structures (cT4b), 5 metachronous colonic resections, 3 major liver resections, 10 multiple hepatic wedge resections, 3 metastasectomies larger than 4 cm, and 2 concomitant resections of other abdominal malignancies were excluded from the study. Finally, 217 patients fulfilled the study criteria and were therefore included. One hundred fifty-three (70.5%) patients underwent an open rectal resection while 64 (29.5%) were operated on by laparoscopy.

### Baseline variables before matching

No significant differences were observed between groups in terms of age, sex, ASA score, BMI, and comorbidities (Table 1). Overall, most of the tumours were located lower than 10 cm from the anal verge with no differences between ORR and LRR groups (62.7% vs. 60.9%; *p* = 0.641). Larger tumours were operated in the open group compared to laparoscopy (3.7 ± 1.9 cm vs. 3.1 ± 1.4 cm; *p* = 0.03) while preoperative clinical staging was comparable both in terms of T and N stage (Table 1). Sixty-two (40.5%) and 30 (46.9%) patients received neoadjuvant therapy in ORR and LRR groups respectively (*p* = 0.36). Finally, more patients in the ORR group underwent concomitant surgical procedures (29.4% vs. 7.8%, *p* = 0.001) (Table 2). Conversion during LLR occurred in 18 (28.1%) patients: six were converted early during the operation because of adhesive syndrome, difficulty in the isolation of the inferior mesenteric vessels, or splenic injury during left colonic flexure mobilization with uncontrolled bleeding, and twelve were converted after vessel’s ligation and colon mobilization to pursue mesorectal dissection through a 10–12-cm Pfannenstiel incision.

**Table 1:** Patients’ characteristics before and after propensity score matching analysis

	Before propensity score matching			After propensity score matching		
	ORR	LRR	*p*	ORR	LRR	*p*
	*n* = 153	*n* = 64		*n* = 98	*n* = 50	
AGE (years, mean, ± SD)	66.9 (± 11.2)	66.5 (± 12.7)	0.843	67.4 (± 10.9)	66.3 (± 13.4)	0.664
SEX (*n*, %)			0.650			0.880
M	93 (60.8%)	41 (64.1%)		62 (63.3%)	31 (62.0%)	
F	60 (39.2%)	23 (35.9%)		36 (36.7%)	19 (38.0%)	
BMI (mean, ± SD)	23.3 (± 3.7)	24.2 (±3.8)	0.266	24.2 (± 3.2)	24.2 (±4.1)	0.932
ASA SCORE (*n*, %)			0.865			0.983
1–2	66 (49.7%)	29 (47.5%)		46 (49.5%)	24 (51.0%)	
3–4	67 (50.3%)	32 (52.5%)		47 (50.5%)	23 (48.9%)	
Comorbidities (*n*, %)	68 (51.1%)	24 (47.1%)	0.826	47 (50.5%)	24 (51.1%)	0.858
Type of comorbidities (*n*, %)			0.696			0.357
Cardiovascular	41 (30.8%)	17 (58.6%)		33 (35.5%)	12 (25.5%)	
Respiratory	18 (13.5%)	6 (20.6%)		9 (9.7%)	9 (19.1%)	
Other	9 (6.8%)	4 (13.8%)		5 (5.4%)	2 (4.3%)	
Tumour distance from the anal verge (*n*, %)			0.698			0.536
10–15 cm	57 (37.3%)	25 (39.1%)		34 (34.7%)	21 (42.0%)	
5–10 cm	59 (38.6%)	21 (31.8%)		36 (36.7%)	14 (28.0%)	
0–5 cm	37 (24.2%)	18 (28.1%)		28 (28.6%)	15 (30.0%)	
Tumour size (cm) mean, ±SD	3.7 (± 1.9)	3.1 (±1.4)	0.034	3.6 (± 1.8)	3.2 (±1.4)	0.290
Median (IQR 25–75%)	3(2.4–4.5)	3 (2.0–3.9)		3 (2.2–4.5)	3 (2.2–4.0)	
Clinical T-stage (*n*, %)			0.372			0.999
cT1	21 (13.7%)	10 (15.7%)		9 (9.2%)	5 (10.0%)	
cT2	32 (20.9%)	12 (18.8%)		21 (21.4%)	11 (22.0%)	
cT3	79 (51.6%)	37 (57.8%)		60 (61.2%)	30 (60.0%)	
cT4	21(13.7%)	5 (7.8%)		8 (8.2%)	4 (8.0%)	
Clinical N-stage (*n*, %)			0.736			0.866
cN0	79 (51.6%)	39 (60.9%)		52 (53.1%)	30 (60.0%)	
cN1	45 (29.4%)	15 (23.4%)		28 (28.6%)	12 (24.0%)	
cN2	29 (18.9%)	10 (15.6%)		18 (18.4%)	8 (16.0%)	
Neoadjuvant therapy (*n*, %)			0.364			0.937
Standard RT + CHT	51 (33.3%)	25 (39.1%)		35 (35.7%)	17 (34.0%)	
Short RT	11 (7.2%)	5 (7.8%)		9 (9.2%)	4 (8.0%)

**Table 2:** Perioperative outcomes before and after propensity score matching

	Before propensity score matching			After propensity score matching		
	ORR	LRR	*p*	ORR	LRR	*p*
	*n* = 153	*n* = 64		*n* = 98	*n* = 50	
Operative time (min, mean ± SD)	220.4 ± 60.8	226.9 ± 57.3	0.464	224.9 ± 63.5	230.7 ± 58.8	0.567
Type of resection (*n* pts, %)						
Anterior resection	136 (88.9%)	56 (87.5%)	0.623	83 (84.7%)	44 (88.0%)	0.712
Abdominoperineal resection	14 (9.2%)	8 (12.5%)		14 (14.3%)	6 (12.0%)	
Hartmann	3 (1.9%)	0 (0.0%)		1 (1.0%)	0 (0.0%)	
Associated procedure (*n* pts, %)	45 (29.4%)	5 (7.8%)	0.001	24 (24.5%)	5 (10.0%)	0.144
Cholecistectomy	4 (2.6%)	1 (1.6%)		3 (3.1%)	1 (2.0%)	
Hepatic metastasectomy (1 metastasis)	4 (2.6%)	1 (1.6%)		1 (1.0%)	1 (2.0%)	
Hepatic metastasectomy (2 metastases)	3 (1.9%)	0 (0.0%)		2 (2.0%)	0 (0.0%)	
Liver biopsy	4 (2.6%)	0 (0.0%)		1 (1.0%)	0 (0.0%)	
Right or left annessiectomy	9 (5.9%)	1 (1.6%)		5 (5.1%)	1 (2.0%)	
Uterine myomectomy	1 (0.7%)	0 (0.0%)		0 (0.0%)	0 (0.0%)	
Splenectomy	3 (1.9%)	0 (0.0%)		2 (2.0%)	0 (0.0%)	
Appendectomy	8 (5.2%)	0 (0.0%)		5 (5.1%)	0 (0.0%)	
Meckel’s diverticulum resection	1 (0.7%)	0 (0.0%)		1 (1.0%)	0 (0.0%)	
Colostomy reversal	2 (1.3%)	1 (1.6%)		1 (1.0%)	1 (2.0%)	
Abdominal hernia repair	5 (3.3%)	1 (1.6%)		1 (1.0%)	1 (2.0%)	
Ureteral injury repair	1 (0.7%)	0 (0.0%)		1 (1.0%)	0 (0.0%)	
Ostomy (*n* pts, %)			0.673			0.342
Ileostomy	60 (39.6%)	21 (32.8%)		40 (40.8%)	15 (30.0%)	
Colostomy	24 (15.7%)	11 (17.2%)		17 (17.3%)	8 (16.0%)	
Time to first flatus (days, mean ± SD)	2.5 ± 0.9	1.8 ± 0.9	< 0.001	2.5 ± 0.9	1.8 ± 0.9	< 0.001
Time to first bowel movement (days, mean ± SD)	3.6 ± 1.6	2.8 ± 1.3	0.009	3.5 ± 1.5	2.8 ± 1.3	0.019
Time to soft oral intake (days, mean ± SD)	3.0 ± 1.2	2.0 ± 1.0	< 0.001	3.0 ± 1.3	2.0 ± 1.0	< 0.001
30-days morbidity (*n* pts, %)	34 (23.2%)	10 (19.6%)	0.596	17 (17.3%)	7 (14.0%)	0.502
Clavien-Dindo classification (*n* pts, %)			0.017			0.401
I–II	26 (17.0%)	4 (6.2%)		14 (14.3%)	4 (8.3%)	
III–IV	8 (5.2%)	6 (9.4%)		3 (3.1%)	3 (6.0%)	
Type of complication						
Anastomotic bleeding	0 (0.0%)	1 (1.6%)		0 (0.0%)	1 (2.0%)	
Anastomotic leak	11 (7.2%)	5 (7.8%)		6 (6.1%)	3 (6.0%)	
Intra-abdominal bleeding	2 (1.3%)	0 (0.0%)		0 (0.0%)	0 (0.0%)	
Canalization delay	3 (1.9%)	2 (3.1%)		2 (2.0%)	1 (2.0%)	
Neurological	0 (0.0%)	0 (0.0%)		0 (0.0%)	0 (0.0%)	
Cardiac	2 (1.3%)	0 (0.0%)		1 (1.0%)	0 (0.0%)	
Pulmonary	6 (3.9%)	0 (0.0%)		3 (3.1%)	0 (0.0%)	
Urinary	3 (1.9%)	0 (0.0%)		2 (2.0%)	0 (0.0%)	
Wound infection	3 (1.9%)	0 (0.0%)		2 (2.0%)	0 (0.0%)	
Other	4 (2.6%)	2 (3.1%)		1 (1.0%)	2 (4.0%)	
Length of stay (days, mean ± SD)	11.5 ± 8.3	8.6 ± 7.4	< 0.001	11.0 ± 8.6	8.8 ± 7.3	< 0.001
30-day mortality (*n* pts, %)	1 (0.7%)	2 (3.8%)	0.155	1 (1.0%)	1 (2.1%)	0.625

### Baseline variables and short-term outcomes after matching

After propensity score matching, 98 ORR were compared to 50 LRR. Anterior rectal resection was the most common type of operation performed in both groups (84.7% in ORR vs. 88% in LRR; *p* = 0.71) and a diverting ostomy was chosen in 57 (58.1%) open and 23 (46%) laparoscopic patients (*p* = 0.34). Postoperative outcomes are depicted in Table 2. Time to first flatus was significantly shorter in laparoscopy (2.5 ± 0.9 in ORR vs. 1.8 ± 0.9 in LRR, *p* < 0.001), as well as time to first bowel movement (3.5 ± 1.5 vs. 2.8 ± 1.3, *p* = 0.01) and time to soft oral intake (3.0 ± 1.3 vs. 2.0 ± 1.0 in, *p* < 0.001). Morbidity was similar between groups (18.6% vs. 14.0%, *p* = 0.50) with most of the complications being minor according to the Clavien-Dindo classification (14.4% in ORR vs. 8.3% in LRR *p* = 0.40). Finally, the length of hospital stay was significantly shorter in the laparoscopic group (11.0 ± 8.6 vs. 8.8 ± 7.3 days; *p* < 0.001).

### Pathological and long-term oncological outcomes (Table 3, Fig. 3)

Pathological examination of the resected specimen showed comparable sizes of the tumour (3.6 vs. 3.2 cm; *p* = 0.29), TNM stage (*p* = 0.96), number of retrieved lymph nodes (21.9 vs. 22.3, *p* = 0.96) as well as number of positive nodes between groups. Furthermore, R0 resection was achieved in 89.8% and 90% of cases in LRR and ORR respectively (*p* = 0.56). Oncological adequateness of the resected specimen was confirmed in 86.7% ORR and in 88.0% LRR with no significant differences (*p* = 0.772). In detail, the distal margin was clear for almost every resection both in open and laparoscopy (99% vs. 100%, *p* = 0.47), whereas the circumferential margin was negative in 91.8% ORR and 90% LRR (*p* = 0.70). Finally, complete mesorectal integrity was achieved in 94.9% ORR and in 98% LRR (*p* = 0.365).

**Table 3:** Pathological data before and after propensity score matching analysis

Oncologic data	Before propensity score matching			After propensity score matching		
	ORR	LRR	*p*	ORR	LRR	*p*
	*n* = 153	*n* = 64		*n* = 98	*n* = 50	
pTNM stage			0.598			0.965
y0	12 (7.9%)	3 (4.7%)		3 (3.1%)	2 4.0%)	
I	27 (17.6%)	14 (21.9%)		19 (19.4.5%)	11 22.0%)	
II	38 (24.8%)	17 (26.5%)		28 (28.6%)	13 (26%)	
III	65 (42.5%)	28 (43.8%)		44 (44.9%)	22 (44.0%)	
IV	11 (7.2)	2 (3.1%)		4 (4.1%)	2 (4.0%)	
Retrieved nodes (mean ± SD)	21.5 ± 13.0	20.4 ± 13.6	0.435	21.9 ± 12.8	22.3 ± 13.7	0.960
Positive nodes (mean ± SD)	2.1 ± 4.0	1.3 ± 2.5	0.176	2.3 ± 4.6	1.46 ± 2.7	0.387
Resection margin (*n*, %)			0.246			0.563
R0	139 (90.8%)	57 (89.1)		88 (89.8%)	45 (90.0%)	
R1	10 (6.5%)	7 (10.9%)		8 (8.2%)	5 (10.0%)	
R2	4 (2.6%)	0 (0.0%)		2 (2.0%)	0 (0.0%)	
Oncological adequateness (*n*, %)			0.742			0.772
Yes	134 (87.6%)	55 (85.9%)		85 (86.7%)	44 (88.0%)	
No	19 (12.4%)	9 (14.4%)		13 (13.3&)	6 (12.0%)	
Mesorectal fascia integrity (*n*, %)			0.625			0.365
Complete	146 (95.4%)	62 (96.9%)		93 (94.9%)	49 (98.0%)	
Incomplete	7 (4.6%)	2 (3.1%)		5 (5.1%)	1 (2.0%)	
Distal margin (*n*, %)			0.517			0.474
≥ 1 mm	152 (99.3%)	64 (100%)		97 (99.0%)	50 (100%)	
< 1	1 (0.7%)	0 (0.0%)		1 (1.0%)	0 (0.0%)	
Circumferential margin (*n*, %)			0.462			0.709
≥ 1 mm	141 (92.2%)	57 (89.1%)		90 (91.8%)	45 (90.0%)	
< 1 mm	12 (7.8%)	7 (10.9%)		8 (8.2%)	5 (10.0%)

**Fig. 3:**
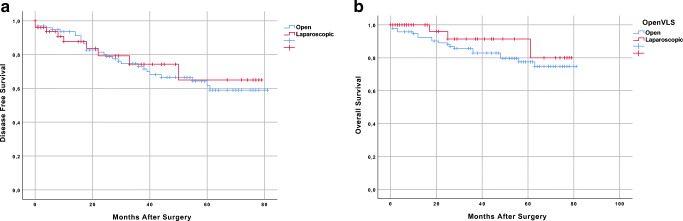
(A) Patient’s overall survival curves according to surgical approach; (B) patient’s disease-free survival curves according to surgical approach

After a median follow-up of 37 (15–61) months, the 3-year actuarial OS rate was 82.9% for open and 91.4% for laparoscopy (*p* = 0.27; Fig. 3). Six patients (6.1%) developed local recurrence following ORR and 2 patients (4.0%) after LRR (*p* = 0.589). The 3-year actuarial DFS rate was 73.1% in ORR and 74.3% in LRR (*p* = 0.82; Fig. 3).

### Oncological adequateness of rectal resections for tumours below the peritoneal reflection (Table 4)

Two subgroups were further analysed according to the location of the tumour below the peritoneal reflection: the middle rectum group (5–10 cm from the anal verge) and the low rectum group (0–5 cm from the anal verge). Patients in both groups underwent a total mesorectal excision and the oncological adequateness of the resection of middle versus low rectal tumours was comparable regardless of the surgical technique (84.0% in middle vs. 76.7% in low rectal cancers; *p* = 0.37).

**Table 4:** Subgroups analysis of the oncological adequateness of middle and low rectal cancers resections after propensity score matching analysis

	Middle rectum			Low rectum		
	(*N* = 50)			(*N* = 43)		
	ORR	LRR	*p* value	ORR	LRR	*p* value
	(*N* = 36)	(*N* = 14)		(*N* = 28)	(*N* = 15)	
Mesorectal fascia integrity, *n* (%)	34 (94.4%)	14 (100%)	0.368	25 (89.3%)	14 (93.3%)	0.663
Distal clearance, *n* (%)	35 (97.2%)	14 (100%)	0.529	28 (100%)	15 (100%)	1.000
Radial clearance, *n* (%)	33 (91.7%)	12 (85.7%)	0.529	23 (82.1%)	13 (86.7%)	0.702
Oncological adequateness, *n* (%)	30 (83.3%)	12 (85.7%)	0.837	21 (75.0%)	12 (80.0%)	0.711
Locoregional recurrence, *n* (%)	1 (2.8%)	0 (0.0%)	0.529	4 (14.3%)	1 (6.7%)	0.458

Comparing ORR and LRR, the oncological adequateness was comparable both in the middle rectum group (83.3% vs. 85.7%, *p* = 0.837) and in the low rectum group (75.0% vs. 80.0%, *p* = 0.71). Furthermore, no statistically significant differences were observed between ORR and LRR patients regarding the distal and circumferential clearance and mesorectal integrity for both middle and low rectum groups (Table 4).

## Discussion

Laparoscopic resection of rectal cancer is still controversial. Despite the short-term benefits that have been widely accepted [14, 17, 22], the oncological adequateness and the long-term survivals following LRR are still debated. Previous randomized clinical trials showed no differences between the open and laparoscopic approaches in terms of oncological adequateness of the surgical specimen; notwithstanding, recently, the ACOSOG Z6051 and the ALaCaRT trial failed to demonstrate the non-inferiority of the laparoscopic technique [13–17]. Indeed, despite long-term results, it showed similar OS, DFS, and locoregional recurrences, higher rates of CRM involvement were shown in laparoscopy [14, 15, 23]. Nevertheless, surgeries not respecting the three pathologic criteria (clearance of the distal and radial margins and integrity of the mesorectal fascia) and therefore failing to achieve oncological adequacy were associated with shorter DFS and increased locoregional recurrence, being the involvement of CRM, the most important parameter influencing survival [24, 25]. In the ongoing debate, the current manuscript demonstrates the oncological adequateness of laparoscopic rectal resection using a propensity score matching analysis that, besides randomisation, represents the best available method to reduce selection bias when analysing the outcomes related to surgical techniques, therefore obtaining high-quality and reliable evidence. No differences in terms of adequacy of the oncological specimen were found between ORR and LRR respecting the oncological criteria in most of the resections and for both approaches. These pathological results eventually translated into comparable OS, DFS, and locoregional recurrence, strengthening the importance of adequate rectal resections in the oncological long-term prognosis of patients. As an additional finding, in our study, we have shown that considering only extraperitoneal tumours, ORR and LRR had a comparable clearance of distal and circumferential margins and mesorectal integrity, defining the resected specimen as oncologically adequate in both approaches. As a matter of fact, during laparoscopic resections for middle and low rectal cancers, advanced technical skills are required to ensure appropriate quality of the operation, especially in the setting of a narrow pelvis, neoadjuvant treatment, large tumours, and/or a high BMI. Indeed, in the above-mentioned scenarios, the adequacy of the resected specimen is much more difficult to obtain, eventually requiring technical proficiency to avoid coning effect and ensure the safety of the procedure both postoperatively and oncologically [14]. Notwithstanding, according to our results, LLRs could be considered oncologically adequate even for middle and low rectal tumours. Robotic surgery and transanal total mesorectal excision (TaTME) were recently developed to overcome some technical issues of standard laparoscopy during technically demanding resections, especially in middle and low rectal resections. The robotic approach seems to ensure wider distal margins as compared to LRR whereas TaTME is helpful to achieve a wider CRM and a complete grading of mesorectal quality [26–32]. Despite this, the ROLARR trial has recently demonstrated that robotic-assisted surgery does not confer an advantage over pure laparoscopy [28] whereas TaTME was recently halted in Norway as part of a national policy because of the early and high recurrence rates [33]. Therefore, emphasis and further evidence should still be encouraged in the setting of laparoscopy as it is surely the most used and widely available technique nowadays.

Minimally invasive resections have been shown as beneficial in terms of postoperative outcomes in different surgical settings [34–36]. Even in colorectal surgery, short-term outcomes are significantly improved when compared to open surgery [14, 17, 22]. First flatus, bowel canalization, and soft oral intake happened significantly earlier in LRR in our study and hospital stay was significantly shorter [14, 17, 22]. No significant differences in terms of postoperative morbidity were observed between groups; nonetheless, ORRs were associated with higher rates of minor complications especially considering wound infections, pneumonia, and urinary tract infections. Larger incisions and slower recovery after surgery are surely contributing and probably affecting both patient’s postoperative period and hospital discharge. In this setting, the role of enhanced recovery after surgery further improves the short-term results of minimally invasive techniques and should therefore be encouraged [37–39].

While laparoscopy currently represents the standard approach for the treatment of right and left colon cancers, resections of the rectum are technically demanding and require an appropriate learning curve, selection of patients, and referral centres. Comparing the two populations of our study before propensity score matching, patients undergoing laparoscopic or open rectal resections were overall comparable in terms of demographics and clinical presentation. Certainly, the LRR group counts fewer patients as a result of the learning curve of a relatively novel technique, but the absence of differences in the unmatched cohorts suggests that selection bias in our experience was not significant even before correction. In our centre, indeed, we tend to approach all rectal tumours by laparoscopy, excluding only cases in which tumour invades surrounding structures (T4b) or in which an associated major procedure is preoperatively planned (i.e. metastatic disease requiring contemporary liver resections). Notwithstanding, we still matched the groups using propensity score analysis to correct for undetectable selection bias eventually improving the statistical power of the study.

This study has some limitations mainly being the retrospective fashion and the relatively small sample size. The study was limited to the most recent 5-year period to avoid bias induced by the learning curve effect. Indeed, in 2013, the 4 surgeons were already all experienced in the laparoscopic colon and rectal resections reducing the bias of surgical technique. Furthermore, they independently performed both open and laparoscopic procedures.

## Conclusions

Laparoscopic surgery for rectal cancer is associated with good postoperative results. Compared to the open approach, oncological adequateness of the resected specimen is respected, even in the setting of middle and low rectal tumours eventually ensuring long-term survivals. Surgeons in high-volume centres should be encouraged to perform more laparoscopic resections in order to provide improved postoperative recovery maintaining safe long-term survival.
